# Balancing act: exploring work-life balance among nursing home staff working long shifts

**DOI:** 10.1186/s12912-024-02165-8

**Published:** 2024-07-23

**Authors:** Kari Ingstad, Gørill Haugan

**Affiliations:** Faculty of Nursing and Health Science, Nord University, Pb. 93, Levanger, 7601 Norway

**Keywords:** Long shifts, Nursing home staff, Shift work, Healthcare professionals, Nursing homes, Family life, Shift schedules, Job satisfaction, Work hours, Work-life conflict

## Abstract

**Background:**

Nursing home staff often face challenges in achieving a satisfactory work-life balance, particularly because of the nature of shift work. While long shifts offer extended periods off work, their impact on the delicate balance between work and leisure remains understudied in the context of nursing homes. This study investigated the experiences of nursing home staff in Norway working long shifts lasting 12–14 h and their perceptions of the balance between family life and work.

**Methods:**

Eighteen nursing home staff members were interviewed following a semi-structured qualitative approach. The participants worked in three types of long shifts and provided insights into their experiences, addressing issues such as work hours, shift patterns, and work-family balance.

**Results:**

The study revealed four main categories: (1) impact of long shifts on family life—the highs and lows; (2) maximizing time off with long shifts; (3) reducing job stress with long shifts; and (4) full-time work leads to predictable hours and stable income. The participants emphasised the distinct separation between work and leisure during long shifts, acknowledging limited social life during working periods but appreciating extended periods off. Family life posed challenges, especially with young children, but the participants found benefits in the longer periods of family time during days off. Longer rest periods and reduced commuting time were perceived as advantages of long shifts, contributing to better sleep, reduced stress and overall well-being. Long shifts also allowed for more predictable working hours and income, supporting a stable work-life balance.

**Conclusion:**

Balancing work and family life involves more than just the number of hours spent at work; it also encompasses the quality of those hours both at work and at home. Our findings underscore the complex interplay between work and family life for nursing home staff working long shifts. While challenges exist, benefits such as extended time off, improved sleep, reduced stress, and predictable working hours contribute positively to their work-life balance. Long shifts in nursing homes offer a unique perspective on achieving work-life balance, revealing both the challenges and advantages inherent in such schedules. Understanding the experiences of nursing home staff in this context can inform future innovations in shift scheduling, promoting a more balanced and sustainable work environment for healthcare professionals. For some healthcare staff, extended shifts can lead to a better work-life balance.

**Supplementary Information:**

The online version contains supplementary material available at 10.1186/s12912-024-02165-8.

## Introduction

Nursing home residents require round-the-clock care, necessitating shift work for staff. On the one hand, shift work may be perceived as flexible; for example, staff can work compressed hours and have longer periods of time off, or they can choose to work nights. On the other hand, shift work can be a negative experience because spare time that differs significantly from that of the majority may have limited social value [1]. A good work-life balance is important to health professionals’ health and quality of life (QOL). Therefore, shift work should be planned to facilitate a sound balance between work and leisure.

In Norway, day-work normally entails 37.5 h a week, while shift-work involves fewer working hours, varying from 33.6 to 35.5 depending on the amount of evening, night, and Sunday work. The Norwegian health system mostly includes timetables of day-, evening-, and weekend-shifts; several schedules also include night-shifts, while some positions only involve night-shifts. Nevertheless, only three per cent of registered nurses (RNs) in Norwegian hospitals work long shifts (12 h or more), while this figure is considerably higher in other comparable countries, such as Finland (8%) and Denmark (12%) [2]. In Norway, the most common working pattern for nurses is five 6–8-hour shifts and work every third weekend [3, 4]. In regions beyond Scandinavia, the healthcare sector employs diverse shift schedules. While a three-shift pattern with two 8-hour day shifts and a night shift remains a common model, many countries, including Ireland, Poland, the USA, and increasingly, the UK, have adopted long shifts lasting 12 h or more as part of a two-shift system [5, 6]. Shift systems can vary significantly across countries depending on shift length, rest periods, breaks, the number of consecutive shifts, whether the system follows a two-part or three-part rotation, and other such factors [7–9].

The organisation of shift schedules in Norwegian nursing homes has led to a significant number of health professionals working part-time. This results from the necessity to manage the staff rota, with many part-time employees needed to fulfil weekend staffing requirements. Among municipal healthcare staff working shifts in Norway, only 32 per cent work full-time [10]. Short shifts may contribute to a more stressful and hectic work experience [11]. A Dutch study indicated that individuals working more than 30–40 h a week experienced less stress than those working fewer hours [12]. Qualitative research must be conducted to examine the reason for this more closely.

Re-evaluating shift schedules must adhere to relevant laws and agreements. Most countries have such regulations [13]. In Norway, employees are required to have at least 11 h of continuous off-duty time within each 24-hour period, with work shifts followed by a daily rest period. Furthermore, employees must have at least 35 h of continuous off-duty time every seven days. Employers and employees` elected representatives in undertakings who are bound by a collective pay agreement may agree in writing to deviate from these requirements [14]. In Norway, an employee who has worked on a Sunday or a public holiday must have the following Sunday or public holiday off. However, employers and employees can agree in writing to a work schedule that ensures employees are off duty, on average, every other Sunday and public holiday over a 26-week period [14]. Furthermore, nursing personnel in Norway typically work a maximum of every third weekend. However, this arrangement is controversial as one-third of the staff who are scheduled to work cannot meet the staffing requirements for weekends, leading to a high reliance on temporary or part-time employees [15].

The optimal arrangement of shift schedules remains unclear [16]. Continuous innovation in shift organisation is necessary to maximise benefits for patients, employers, and employees. This study aimed to investigate the experiences of nursing home staff in Norway regarding the balance between family life and long shifts. Throughout the study, the term ‘long shifts’ refers to a working schedule based on shifts lasting 12–14 hours.

### Work-life balance

Work-life balance can be defined as ‘the individual perception that work and non-work activities are compatible and promote growth in accordance with an individual’s current life priorities’ [17]. Achieving a healthier work-life balance not only enhances job satisfaction, performance, and commitment to the organization but also contributes to overall life and family satisfaction [18]. Furthermore, maintaining a balanced work-life dynamic is linked to lower levels of stress-related outcomes, including psychological distress, emotional exhaustion, anxiety, and depression [18]. A healthcare staff who works beyond the normal working day may find it particularly challenging to balance work and family obligations because they must be at work when kindergartens and schools are closed. However, working long shifts can reduce the impact of shift work on family and social life because longer shifts mean fewer days at work [9]. Studies on this are inconclusive. On the one hand, studies show that nurses often prefer long shifts as they improve work-life balance [19, 20]. The extra days off are also often mentioned as the reason for a preference for long shifts [21, 22]. However, work-life balance is also positively rated by nurses working 8-hour shifts [20].

Time is a limited resource for most families with children, who face competing demands on their time. However, perceptions of a good balance between work and family may differ among nurses. Whether this relationship is perceived as conflictual will be influenced by individual factors, macrostructures, and work organisation [8, 22]. Many nurses care for children or ageing parents and provide wages and benefits critical to their families’ basic needs [22]. Exploring how the organisation of working hours can maintain work-life balance is important because this can reduce turnover and nurses’ intention to leave [23]. Exploring this within the municipality health service will be particularly significant because municipalities often lack the depth of professional expertise found in hospitals, and part-time employment is more prevalent in municipal settings than in hospitals across Norway [24]. As municipalities have assumed numerous responsibilities previously managed by hospitals, including handling a wide array of advanced and intricate tasks [25], ensuring optimal working conditions, and adhering to working time regulations can contribute to staff well-being and development. This, in turn, can facilitate retention within the services [26], which is crucial given the expected increase in the shortage of healthcare professionals in the years to come [24].

Most previous studies on the arrangements of nurses’ working hours and work-life balance have been conducted in hospitals [16]. To the authors’ knowledge, no such studies have been conducted in nursing homes. Hence, this study aimed to explore how nursing home staff experience balancing family life with working long shifts.

## Method

The study utilized a qualitative research design with a hermeneutic phenomenological approach to explore the lived experiences of nurses balancing family life while working long shifts. This approach guided the researchers to deeply engage with participants’ lived experiences and derive meaningful, contextually grounded interpretations [27]. The study included 18 individual semi-structured qualitative interviews. All the participants worked in nursing homes in three different types of long shifts. Two worked extremely long shifts: 14-hour shifts on seven consecutive days followed by two weeks off. Fourteen worked 12–14-hour shifts for 3–4 days with one week off between each work period. In these nursing homes, these were the only types of shifts available to the ward staff. Two of the participants worked long shifts every fourth weekend but otherwise worked 6–8-hour shifts. Among the informants, three did not have children, seven had children living at home aged 3 to 16, and eight had children who had moved out. Of those eight, seven regularly looked after their grandchildren. Table 1 presents participant characteristics.

Work in nursing homes encompasses a broad range of responsibilities, from providing practical assistance and psychological support to delivering advanced medical treatments, such as administering medication and wound care. Most patients are elderly individuals with various chronic illnesses, disabilities, or frailties, and many suffer from dementia. Both RN and assistant nurses in Norwegian nursing homes address patients’ basic needs, including personal hygiene and nutrition. Furthermore, RN handle medications safely, monitor their effects, and have the authority to administer certain medications independently. Additionally, both registered and assistant nurses in Norway often perform tasks that do not necessarily require their level of education but are well within the competence of the assistants [26].

**Table 1:** Participant characteristics (*N* = 18)

Age	27–63 (Average age: 47)
Females/Males	17/1
Percentage of full-time position	70–79%: 3 80–89%: 6 90–100%: 9
Registered nurse/Assistant nurse*	11/7

*Assistant nurses attend vocational schools for their training

### Sample and procedure

Strategic sampling was employed to ensure that participants could shed light on the research question. Inclusion criteria were that participants worked in a nursing home with shifts of at least 12 h. The first author gathered information from the media and employee organisations about nursing homes that use long shifts. The managers of these nursing homes were contacted and asked to pass on letters to potential participants working long shifts; they were asked to find a broad sample of participants in terms of age, sex, and attitude. The participants were contacted by phone one week after receiving a written invitation. Eighteen participants were contacted, and they all attended the interview. They worked in four nursing homes in four different counties. The interviews took place at the nursing homes where they worked. Based on previous research in the field, an interview guide was prepared for the interviews. The interview guide was developed specifically for this study, including topics such as working hours, shift patterns, perceived workloads, and work-family balance. The study interview guide is provided as supplementary file 1.

### Analysis

The analysis draws on Kvale’s approach to phenomenological hermeneutic analysis, which is characterized by a systematic and reflexive process that integrates a deep understanding of participants’ experiences with ongoing interpretation and critical reflection [28]. This involved a dialectical movement between the parts and the whole, employing a continuous back-and-forth process based on the hermeneutic circle to achieve progressively deeper insights into meaning [27, 28]. Our comprehension evolved throughout the interviews, data processing, and dissemination processes. Specifically, during the interviews and transcription process, analytical and theoretical ideas, as well as noteworthy statements, were frequently noted. The initial examination of the text provided a description of the staff’s experiences of working long shifts, offering a preliminary interpretation. Subsequent in-depth analyses yielded a more profound understanding of the text’s content. For example, we gained insight into how employees’ experiences of long shifts facilitated a clearer separation between work and private life. Particularly, long shifts provided longer continuous periods of time off, making it easier to fully disconnect during this time. This reflects what Gadamer described as reaching a new horizon [27]. Meaning categorisation was employed as an analytical tool, generating categories and sub-categories during the analysis [28] (see Table 2). Based on the interviews and the interpretations derived from them, key attributes of the studied phenomenon were identified, which allowed us to capture nursing home staff’s experiences of balancing family life and work while working 12–14 h shifts. Throughout the analysis, the categories were changed, abstracted, and adapted to the data. The coding process aimed to reduce the amount of data and gain a clearer idea of the topics emphasised by the participants.

**Table 2:** Examples of categorisation

Category	Sub-category	Statements
Impact of Long Shifts on Family Life: The Highs and Lows	Intensive periods of work	*You’re totally antisocial the week you work, it’s just work and sleep, that’s all you have time for.*
		*The four days you’re at work you don’t have much in terms of privacy.*
	Periods with plenty of time off	*I have so much spare time… I can go for walks in the mountains, I can study.*
	Periods away from family that are worse than others	*That’s the worst thing. This year my shift falls on the 23rd of December, Christmas Eve, and Christmas Day, and that’s not much fun.*

### Ethics

The possible consequences of an interview study should be assessed in terms of harm to the interviewees [28]. Participation was voluntary, and participants could withdraw at any time. All participants provided written consent. Little sensitive information emerged during the interviews, and none of the participants withdrew. The participants were happy to contribute to a research project that shed light on their experiences of working long shifts.

## Trustworthiness

Ensuring the trustworthiness of qualitative studies involves maintaining credibility, dependability, confirmability, and data transferability [29]. In this study, the authors’ experiences as nurses and familiarity with the context bolstered trust and enriched data collection. Continual review and detailed analysis ensured data dependability, aligning the findings with raw data to enhance reliability. To ensure confirmability, the study described participants’ experiences in detail, thereby making the findings meaningful and transferable. The researchers relied heavily on raw data during analysis to prevent data loss and validated the findings by referencing supporting research while addressing conflicting evidence.

## Results

A work schedule of long shifts entails employees working for many consecutive hours, with extended periods of time off between each work period. The duration of time off is linked to the length of the shifts and the number of consecutive shifts worked. Employees with many consecutive long shifts will enjoy more extended periods of time off. This study delineates how long shifts may impact staff experiences of balancing work and leisure. The findings are presented in four categories:

1. Impact of long shifts on family life—the highs and lows.
2. Maximizing time off with long shifts.
3. Reducing job stress with long shifts.
4. Full-time work leading to predictable hours and stable income.

### Impact of long shifts on family life—the highs and lows

Working a 12-hour shift means that much of one’s waking hours are spent at work. Long workdays, combined with extended time off, create a distinction between work and leisure. This approach has clear advantages, but it also comes with certain challenges. In particular, someone who works long shifts has considerably less time and energy for other activities during work periods. In this study, two participants described how working long shifts affected their social life during these times:

*You’re totally antisocial the week you work, it’s just work and sleep, that’s all you have time for.* (5)

*The four days you’re at work you don’t have much in terms of privacy.* (4)

Long shifts can pose challenges because children’s schedules often do not align with such extended work hours. Both kindergartens and schools typically close at 4 or 5 pm. Consequently, parents working long shifts may encounter difficulties harmonising this schedule with family life. However, family situations and obligations vary, including different family constellations. Parents of young children may require assistance in picking up their children from kindergarten and school and taking care of them throughout the evening. Furthermore, this implies that during working periods, parents may have limited time with their children. The advantage lies in the longer periods off, allowing for ample family time:

*If you’ve got a family, you don’t see your children for four days, they’ve gone to bed when you get home, and you only see them briefly in the mornings. But it’s the same when you work normal shifts, you have late shifts then too, and parents with children in kindergarten and school can’t pick them up and then go on a late shift, you must have someone else to pick them up.* (4)

*I fully understand my work situation, how we share the housework and so on. So, for us, it works fine. But I notice that our youngest boy isn’t happy when I start work, because then I’ll be away so much. But when we talk about it and say that I’ll have a whole week off, then he says yes, because that means so much. Then things are ok.* (16)

The need for babysitting in the evenings and on weekends is a common requirement for anyone with children working shifts, regardless of the shift-work organisation. However, being away from one’s family almost the entire day for several days poses a specific challenge associated with long shifts, necessitating others to willingly care for the children during working periods. However, long shifts also offer extended periods of leisure time, providing opportunities for activities that may be impractical with traditional shift-work. Some participants believed that the benefits outweigh the disadvantages. Having several consecutive days off allows for travel, caring for grandchildren, hiking, and complete relaxation. The extended periods of free time were highlighted as a particularly positive aspect of working long shifts:

*If we must go back to traditional shifts, I think I might retire. Because this shift really suits me. I can travel, I love travelling. I can look after my grandchildren, and I have time to arrange things if there’s something special.* (2)

Long shifts encompass intensive work periods and extended periods of time off, which are distributed systematically throughout the year. Compressed work periods can be an advantage at times, but if they coincide with public holidays, nurses naturally find it annoying. As one participant explained:

*That’s the worst thing, this year my shift falls on the 23rd of December, Christmas Eve, and Christmas Day, and that’s not much fun. But last year it was another team that had Christmas, and then I had time off until New Year’s Day, so this year it’s our turn.* (10)

Some periods away from the family are worse than others. If work periods coincide with Christmas or Easter, it can be particularly frustrating to work long shifts because one must be at work all day for several days while most other people spend time off celebrating the holidays.

### Maximizing time off with long shifts

Long shifts mean intense work periods. The total of 35.5 h per week is completed in under five days. Consequently, staff who work long shifts have longer periods of time off than those who work traditional shifts. Longer time off means more possibilities and flexibility. One participant said:

*I have so much spare time… I can go for walks in the mountains, I can study.* (1)

With regular shifts lasting 7–8 h, having only 1–2 days off in a row is common. Simultaneously, when the work involves significant caring responsibility and emotional strain, many people may think about their job even after the shift is over. Longer shifts result in longer continuous periods of time off, allowing for a greater degree of disconnection from work.

*I used to feel like I was at work all the time, you did day shift, evening shift, evening shift, day shift. You had one day off before the weekend and one day after. That wasn’t so good for me. Now I’m so happy with this arrangement, you can’t imagine. You can plan a lot more, I can travel, you can do so many things. I couldn’t do those things when I was doing day and evening shifts.* (3)

For healthcare professionals working long shifts, the length of time off depends on the shift duration and the consecutive shifts worked. Typically, they can enjoy 3–8 days off consecutively. Extended periods of time off facilitate detachment from work, allowing for complete relaxation. In other words, the scheduling of long shifts facilitates detachment from work during off periods, suggesting reduced stress and improved recovery during leisure time.

### Reduced job stress with long shifts

With 12-hour shifts, work can be organized differently than with traditional 7–8-hour shifts. The extended duration allows for more consecutive hours at work, facilitating innovative ways of task allocation. Contrastingly, 8-hour shifts impose tighter time constraints for completing tasks before the next shift commences in the evening. However, the flexibility of 12-hour shifts permits task distribution in new and creative ways, potentially reducing stress levels. Additionally, long shifts ensure work continuity throughout the day, enhancing workflow efficiency. Two participants described it as follows:

*I have a clear view of my patients from morning to evening. (8)*

*There’s less stress with long shifts. If it’s showering, wound care, or similar tasks, we can do them in the afternoon if the morning is busy. (2)*

Twelve-hour shifts involve two handovers per day instead of the usual three in traditional shift schedules. With fewer shifts, less time is spent changing shifts, ensuring greater continuity throughout the day and longer intervals between shifts. When shifts are 12–14 h long, there will be at least 10–12 h between each shift, which helps mitigate the challenges of quick returns. The participants highlighted the longer rest periods between shifts as an advantage of long shifts:

*You used to have more changing from late shift to early shift, then you finished at quarter past ten and had to be back here at 7.30 the next morning, which made me sleep worse at night. Now when I get home at half past eight, I have a lot of the evening left so I can relax before I go to bed, and then I sleep better. So, it also means you’re not so tired. When you get home you can sink into a comfortable chair and watch some TV, but when you get home at half past ten and you know you have to get up at half past five, you just think you must hurry up and get to sleep. In fact, I’m not more tired now.* (10)

*It was a bit too much stress to finish at a quarter past ten when you had to be back at work again at half past seven. It was more stressful to sleep then than it is now.* (9)

Short rest periods between shifts can cause stress owing to worries about getting enough sleep. Some of this stress can be avoided with long shifts because of the longer time to rest and sleep between shifts. The length of shifts also affects the number of days at work and time spent commuting. Long shifts involve fewer days at work than traditional shifts:

*I couldn’t stand it if I had to do two shifts in the usual way, because I have an hour’s drive to get to work, and then I’d come here and work seven hours, then I’d drive home and be at home just a few hours before I went back to work for seven more hours. No, I couldn’t stand that, it would be too hard on me.* (3)

Long shifts mean reduced time spent commuting. Hence, long shifts are advantageous to those travelling from afar; less time spent commuting means more free time.

The participants perceived that compressed working hours, coupled with longer periods off, facilitated a clearer work-life separation compared with traditional 6–8-hour shifts. Social life during the working period was extremely limited. In this manner, participants working long shifts experienced a distinct separation between work and leisure:

*I have so much spare time, and when I’m at home I don’t have to think about things at work. That’s the best part of it. I can more easily separate work and time off. I’m not always at work, and when I’m at home I can do other things. (1)*

*You have less stress with a schedule like that. When you’re at work, you’re at work, and when you’re at home you have time off. Your stress level goes down. (4)*

A clear distinction between work and leisure time may have both advantages and disadvantages. On the one hand, it fosters uninterrupted continuity during work hours. On the other hand, it results in extended periods of time off, leading to breaks in continuity. For instance, nurses may go more than a week without seeing a patient, necessitating significant catch-up upon return to work.

*I feel like I’ve lost track of things after a week off. That’s why we’ve started implementing this one-day overlap. (9)*

This discontinuity can pose challenges, particularly for employees working in fixed teams. However, implementing overlap periods between teams, where employees from different shifts coincide, can mitigate this issue.

### Full-time work leads to predictable hours and stable income

6–8-hour shifts and work every third weekend represent the traditional scheme in Norway, causing many employees to work part-time. In contrast, long shifts make it possible to work in only one ward, supporting continuity and thereby enhancing patient safety while facilitating a stable and positive working environment. Moreover, long shifts encourage staff to take on larger percentages of full-time positions. Consequently, working full-time or almost full-time provides predictable working hours and a reliable income, contributing to a more stable situation both financially and socially:

*If I can’t carry on in this kind of shift work, I’ll have to go back to a 60% job. And then I don’t know if I want to continue. Because then I’ll have that hectic life with short shifts and a lot of driving and never any free time. Because then I’d have to start doing more work to make ends meet and have ok finances.* (3)

*I used to work on three wards to reach a full-time position, but now I feel a sense of belonging to this ward. I feel more self-confident, I feel that I know this field for this group of patients.* (7)

Several participants, particularly assistant nurses, increased their full-time job percentage after changing to long shifts, and all of them had at least a 70 per cent position. A high percentage makes it easier to understand when to go to work and when to take time off. It also ensures a predictable income, eliminating the need to be on the lookout for extra shifts to make ends meet. Furthermore, the participants were pleased that they now only worked in one ward. Having a high percentage of a full-time position in a single ward provides continuity, greater professional confidence, improved collaboration, a sense of belonging, and overall well-being—all factors known to support patient security.

## Discussion

The association between work and family life is frequently examined through the lens of work-life balance or work-life integration [30]. Work-family balance entails aligning work and non-work activities to support personal growth in line with an individual’s current life priorities [17]. It transcends mere job satisfaction, influencing overall life and family contentment while also reducing stress-related outcomes [18]. Thus, work-life balance is not solely dependent on the number of hours spent at work; it also encompasses how individuals feel both during work hours and when they are off duty.

Maintaining a balance between work and leisure poses particular challenges, especially for healthcare professionals working in shifts [8, 23, 31]. Patients require round-the-clock care, necessitating staff to work during hours when schools and kindergartens are closed and when others typically have time off. Hence, for individuals working outside conventional working hours, designing shifts that optimize work-life balance for as many staff members as possible is crucial. Moreover, laws and agreements must prioritise both patients’ needs and those of employees for work-life balance. In Norway, debate is ongoing about whether existing regulations adequately address this issue, given that current mandates, such as working only every third weekend, result in numerous part-time positions [26]. A proposed solution for increasing full-time employment is the adoption of long shifts [32]. However, whether long shifts can effectively enhance work-life balance remains uncertain. The present study explored the firsthand experiences of nursing home staff engaged in long shifts, specifically focusing on how they navigate the balance between work responsibilities and personal time.

The results showed that working long shifts supports a clearer separation between work and leisure time compared with the ‘normal’ working schedule. During working periods, nurses’ focus is on work, while during non-working periods they have plenty of time to relax and do other things. Long shifts involve compressed working hours accompanied by a considerably limited social life along with little time to spend with children and other family members. Children may be unhappy about hardly seeing their parent for several days. Furthermore, when one’s work periods coincide with Easter, Christmas, and other public holidays, working can be tiresome and make one feel that one is losing out. Nevertheless, the participants in this study felt that these disadvantages were outweighed by the advantages of working long shifts. Long shifts provide extended periods of time off, allowing healthcare professionals to pursue leisure activities, spend time with family, or engage in hobbies. This flexibility can enhance overall well-being, which is not always possible with traditional shifts. Long shifts include a longer break of 10–12 h between shifts supporting better sleep and recovery, whereas the traditional shift schedule may provide only 8–9 h between evening and day shifts, causing more stress and insomnia. The participants underlined the benefit of having a longer period between finishing work in the evening and leaving for work in the morning; the extra time made it easier to relax and calm down after the first shift and resulted in better sleep. ‘Quick returns’, involving a short break between evening and day shifts, are more common in Norway than in other countries. In the Norwegian context, as many as 64 per cent of hospital nurses have more than 13 evening-to-day shift transitions per year, while in Finland the figure is 47 per cent and in Denmark 16 per cent [2]. Such quick returns can worsen the work-life balance [33] and cause more stress. This is considerably less of a problem with long shifts because of the longer time off between the shifts.

Some participants saw it as an advantage that long shifts mean less time spent commuting because of fewer days at work. Long shifts of 12.5 h result in 133 days at work per year, and 232 days off. Shifts of 7.5 h will mean 222 days at work and 143 days off, while six-hour shifts will involve 277 days at work and 88 days off (see Table 3). Fewer days at work lead to less commuting time and will be particularly helpful for staff with a long journey between home and work.

**Table 3:** Overview of the number of attendance days by shift length

Shift length in hours	Number of attendance days during 1 year	Number of days off in a year (including 5 weeks of vacation)	Average number of days off per week
7.1	235	130	2
9.0	185	180	3
12.5	133	232	4.2
14	119	246	4.5

Balancing work and family life also implies predictable working hours and pay.

In municipal health and care services in Norway, 57% of nurses are employed on a full-time basis, and 28.5% of assistant nurses follow suit [24]. One might expect that part-time work makes it easier to combine work and family/leisure time. However, studies show that this is not necessarily the case. Healthcare workers in part-time jobs report an equal or greater degree of work-family conflict than those working full-time [12, 31]. The way work is organised seems to be more important for a good work-life balance than whether one works part-time [31, 34]. This may partly be because many part-time staff work more than their fixed hours, that is, they work extra shifts to get a living wage. Moreover, on days which basically are ‘my-day-off’, they must be willing to work, often at short notice. Part-time workers may be considered ‘second-class workers’ who are subject to stress and strain in the form of inconvenient shifts, lack of control, and no clear work schedule [31, 35, 36]. Working part-time can result from the employer’s need for part-time employees as much as the employee’s preference for part-time work [37].

A part-time job along with a search for extra shifts means an unreliable income and unpredictable working hours and time off [3, 36]. Lack of control over one’s working hours causes stress and is an additional work-related burden [38]. This study highlights that long shifts, particularly for assistant nurses, offer positions that are (almost) full-time, with more predictable working hours and income. Other studies have shown that long shifts are a way of organising work that gives employees more predictable working hours, leisure, income [32], and a better balance between work and family life than ordinary shifts [9, 39]. Many employees enjoy shift-work; evidence shows that those who work long shifts and jointly planned shifts (where staff have a say in their shifts) express the greatest satisfaction with shift-work [39].

Shifts are not solely about meeting employees’ preferences and requirements; they are equally important for maintaining high-quality services for patients. This study explored the experiences of employees. It highlighted that increased full-time positions and staff continuity during long shifts offer greater flexibility and reduce job stress throughout the working day, facilitating efficient handling of necessary tasks. However, extended periods off work can disrupt this continuity, suggesting the need to implement overlap periods between teams to ensure uninterrupted service continuity. Additionally, when nurses work long shifts with extended time off, it may affect their ability to supervise students. If the students are not working long shifts themselves, it becomes challenging to provide adequate follow-up when nurses have extended time away from work.

Working hours consist of several dimensions: structural, quantitative, and qualitative. Important factors may be the length of shifts, working hours per week, opportunity to take breaks, shift rotation, working environment, self-perceived competence, well-being, control, and work intensity [8]. Working hours represent both a key economic and cultural category, as well as a social category. The notion of balance has been criticised because it assumes that individuals should achieve an appropriate distribution of hours between work, family, and leisure [40]. A related concept is work-family conflict, which refers to difficulty in combining work and family roles [41]. The term ‘work-family conflict’ seems useful since staff experiences of combining family and employee roles are fundamental. This study has shown that staff who work long shifts experience a conflict between work and family but still find more advantages than disadvantages to this type of shift schedule. For example, many employees prefer not to work over weekends. Nevertheless, this is mostly impossible to avoid in the health sector, as patients also need care and treatment at weekends. Within a long shift schedule, staff not only work more hours at weekends during their compressed work periods but also have more weekends off [11]. Employees working longer shifts on weekends benefit from more weekends off. When employees are satisfied with this arrangement, it highlights that work-family balance is influenced not only by the number of hours worked but also by how those hours are organized.

New generations of employees often want to participate fully in the working life, which calls for employers to draw up shift schedules that are adapted to a culture of full-time work [36]. The current lack of health professionals indicates a need for fundamental changes to the shift schedule to make it compatible with full-time jobs, which allow a predictable salary and work-life balance. Efforts to create a sustainable shift rotation system based on full-time positions must enable a balanced rhythm between recovery and work [36, 42].

### Strengths and limitations

Long shifts are uncommon in nursing homes across Norway, with only a few establishments adopting this system. Consequently, qualitative in-depth interviews serve as a suitable method for understanding how employees perceive the impact of long shifts on their work-life balance. However, considering the limited availability of long shifts, relatively few healthcare workers have the opportunity to experience them. All the participants in this study willingly applied for positions involving long shifts, despite having the option to opt for departments with traditional shifts. In this regard, had these employees been compelled to work such shifts, additional insights into long shifts could have been obtained.

## Conclusion

Balancing work and family life isn’t just about the number of hours spent at work; it’s also about the quality of those hours both at work and at home. Whether working long or short shifts, the total hours may be the same, but long shifts can feel less stressful while on duty. Additionally, longer consecutive periods of time off, as seen with long shifts, provide a different experience compared to having more daily time off with short shifts. This study contributes new insights into how nursing home employees experience work-family balance when working long shifts. The findings indicate that long shifts better separate work and leisure than traditional shifts. During working periods, social life and hobbies are significantly limited, providing little time for children and other family members. One’s entire focus is on work during these periods. However, during the off-work period, one can relax and do completely different things without thinking about work. Some participants considered this a good way of organising their work-life, providing a better balance between work and leisure. Long shifts result in less time spent commuting because fewer days are spent at work and eliminate quick returns, as the rest period from finishing work in the evening until starting again in the morning is longer. Furthermore, long shifts provide staff with more full-time or almost full-time jobs, which provide predictable pay, working hours, and leisure time. Healthcare workers may have different wishes and needs, and their family situation will vary. Similarly, the extent to which long shifts improve employees’ work-life balance will vary. Nonetheless, this study showed that long shifts enable a clearer separation of work and leisure, followed by less stress, better sleep, and recovery. Logically, reduced stress and enhanced continuity in patient care are positive side effects of long shifts. Balancing work and leisure can be particularly challenging for shift workers, making it important to draw up shift schedules that provide the best possible work-life balance.

### Electronic supplementary material

Below is the link to the electronic supplementary material.

Supplementary Material 1

## Data Availability

The data will be available from the corresponding author on request.
