# Supplementary material for: Balancing act: exploring work-life balance among nursing home staff working long shifts
Source: BMC Nurs. 2024 Jul 23;23:499. doi: 10.1186/s12912-024-02165-8 (PMC11264412; doi:10.1186/s12912-024-02165-8)
Supplement: Supplementary file 1 — Supplementary Material 1 [file 12912_2024_2165_MOESM1_ESM.docx]

**INTERVIEW GUIDE - LONG SHIFTS**

***Introduction***

- Brief introduction about myself, the project, and its objectives.
- Assurance of anonymity and clarification on the reporting format.
- Explanation of the interview structure and the right to interrupt at any time.
- Encouragement for honest responses. Recording confirmation.

***Theme 1: Background Data***

1. Family Situation:
   - Age, marital status, spouse's occupation/education, children, and grandchildren proximity.
2. Career Path:
   - Educational background, diverse workplaces, full-time/part-time experiences, leaves of absence or career breaks.
   - Current employment percentage and reasons for working part-time/full-time in the sector.

***Theme 2: Working Life***

1. Current Work Schedule:
   - Details on shifts, weekends, hours, department, and desired job size.
2. Breaks and Personal Space:
   - Frequency, activities during breaks, and thoughts on personal space.
3. Process Involvement:
   - Participation in the scheduling process, autonomy, and experiences with different rotas.
4. Working Environment:
   - Relationships with colleagues, cooperation, communication, and the impact of long shifts.
5. Well-being:
   - Factors influencing happiness/unhappiness, stress, sleep, and recovery strategies.
6. Recruitment and Turnover:
   - Relationship between long shifts and recruitment stability, salary, and workloads.
7. Health Impact:
   - Effects on health, ability to work long-term, and comparison between age groups.
8. Ideal Healthcare System:

- Participant's vision for an ideal organization and views on full-time/part-time positions.

11. Sick Leave

- Perceptions on sickness, temporary worker coverage, and correlation with long shifts.

12. Communication Challenges:

- Overlapping shifts, difficulties in restarting after long absences, and preferred communication methods.

13. Service Quality:

- Impact on patient well-being, continuity, and observations regarding errors, especially towards the end of shifts.

**Theme 3 Work-Life Balance**

14. Balancing work and personal life, changes in social life, and division of labor at home.

- Impact on family commitments and how family situations influence working hours.
- Can you tell us a bit about how you manage to combine your work and private life since you started working long shifts? (What do your family say about your rota?)

15. Leisure/Social life

- How do you manage to take care of leisure interests, differently with traditional shifts?
- How has your social life changed (social network)?

18. Labour in home

- Can you say a little about how the division of labor is in your home. Paid work (Do you feel that you are the breadwinner of the family on an equal footing with your partner? Unpaid work (housework: partner, children).
- Has this changed since you started working long hours?

20. Family commitments (caring for grandchildren, parents, spouse).

- Are you expected to be there for your family, how? How do you manage to combine family commitments with working long shifts? What would this be like if you worked traditional shifts?
- Does your family situation affect your working hours/your desire to work/not work long shifts?

**Closing Remarks:**

Participant's thoughts on continuing in this type of rotation and any additional insights.

*End of Interview Guide*
